# Macrophage duality in liver diseases: heterogeneity, plasticity, and the quest for precision therapeutics

**DOI:** 10.3389/fimmu.2026.1814879

**Published:** 2026-04-14

**Authors:** Liwei Chen, Yuhang Chen, Mengxiao Zhao, Xuecheng Yang, Kathrin Sutte, Mengji Lu, Jieliang Chen, Xin Zheng, Dongliang Yang, Jia Liu

**Affiliations:** 1 Department of Infectious Diseases, Union Hospital, Tongji Medical College, Huazhong University of Science and Technology, Wuhan, China; 2 Institute of Infectious Diseases and Immunity, Union Hospital, Tongji Medical College, Huazhong University of Science and Technology, Wuhan, China; 3 Institute for Virology, University Hospital Essen, University of Duisburg-Essen, Essen, Germany; 4 Key Laboratory of Medical Molecular Virology (MOE/NHC/CAMS), School of Basic Medical Sciences, Huashan Hospital, Shanghai Medical College Fudan University, Shanghai, China

**Keywords:** liver diseases, Kupffer cells, monocyte-derived macrophages, macrophage heterogeneity, functional duality, precision medicine, single-cell sequencing

## Abstract

Liver macrophages are master regulators of disease progression and resolution, whose functions transcend the simplistic M1/M2 dichotomy. This review synthesizes cutting-edge evidence to establish a dynamic, subset-centric paradigm for understanding macrophage plasticity. In acute liver injury, we delineate how temporally segregated subsets (e.g., early CCR2^+^ vs. late MerTK^+^ macrophages) sequentially orchestrate damage and repair. In chronic settings—including HBV infection, MASLD/MASH, and fibrosis/cirrhosis—we reveal a complex coexistence of functionally opposed subsets that concurrently drive pathology (e.g., *CD9*^+^*TREM2*^+^ macrophages) and promote resolution (e.g., CD300E^+^ macrophages). This refined understanding underpins a critical appraisal of the evolving therapeutic landscape, which is pivoting from broad immunosuppression towards precision strategies. These include pharmacological targeting of specific recruitment or signaling pathways, adoptive transfer of reparative subsets, and innovative engineered cell therapies (e.g., signal-converting CAR-Ms). By framing macrophage functional duality within the “friend or foe” paradigm, this review provides a conceptual framework for developing subset-specific therapies that harness the beneficial while mitigating the harmful faces of hepatic macrophages. We highlight that the key translational challenge lies in mastering macrophage heterogeneity—requiring resolution of *in vivo* dynamics, cross-species discrepancies, and achievement of durable, context-specific modulation. The future of “macrophage medicine” hinges on integrating spatial multi-omics and advanced imaging to enable spatiotemporally precise interventions, ultimately leveraging the cell’s inherent plasticity for therapeutic gain.

## Highlights

- **Paradigm Shift:** Moves beyond the M1/M2 framework to a dynamic, subset-centric understanding of liver macrophage function in health and disease.
- **Functional Duality:** Macrophage subsets exhibit spatiotemporally specific roles, driving injury or repair in both acute and chronic liver diseases.
- **Precision Therapeutics:** Treatment strategies are evolving towards subset-specific targeting, including pharmacological agents, adoptive cell transfer, and engineered macrophages.
- **Translational Hurdles:** Major challenges include unresolved *in vivo* dynamics, species differences, and ensuring durable phenotypic stability of therapeutic cells.
- **Future Vision:** Spatiotemporally precise “macrophage medicine” will require integration of spatial multi-omics, advanced imaging, and intelligent clinical trial design.

## 1 Introduction

Liver disease represents a significant and growing global public health burden, contributing to over two million deaths annually (1). The pathogenesis of various liver conditions—ranging from acute injury to chronic fibrosis, viral hepatitis, and metabolic dysfunction-associated steatotic liver disease (MASLD)—involves complex interactions between parenchymal damage, inflammatory responses, and reparative processes (2, 3). Within this intricate pathophysiology, innate immune cells play a decisive role in steering disease progression versus resolution (4, 5).

As central sentinels and effectors of the hepatic innate immune system, liver macrophages have emerged as key regulators of liver homeostasis and disease (6, 7). This heterogeneous population exhibits remarkable plasticity and is primarily composed of two origins: the embryo-derived, self-renewing tissue-resident Kupffer cells (KCs) and the monocyte-derived macrophages (MoMFs) recruited upon injury (8). Under physiological conditions, KCs maintain immune tolerance and perform vital surveillance functions. Following hepatic insult, however, MoMFs infiltrate the liver in large numbers and can adopt functionally divergent phenotypes, driving either pro-inflammatory tissue injury or anti-inflammatory repair (9).

Recent advances in single-cell sequencing and other high-resolution technologies have dramatically accelerated the discovery of novel macrophage subsets and states across disease contexts (10, 11). While the traditional M1/M2 polarization framework has been invaluable for conceptualizing macrophage functional duality, it is increasingly recognized as an oversimplification that fails to capture the full spectrum of phenotypes observed *in vivo*. This is particularly evident in the liver, where single-cell technologies have revealed a far more intricate landscape of coexisting, specialized subsets beyond this binary classification (12, 13). However, despite this newfound complexity, critical gaps remain in our understanding of their dynamic phenotypic transitions, lineage relationships, and the spatiotemporal regulation of their dual roles (14–16). A nuanced comprehension of these aspects is essential for developing targeted therapies that modulate specific macrophage functions rather than broadly suppressing this versatile cell type (17, 18). While this review focuses on macrophages, neutrophils also contribute significantly to liver inflammation and repair, and their interactions with macrophages are an important aspect of the hepatic innate immune response.

Therefore, this review aims to systematically synthesize current knowledge on the identity and function of key macrophage subsets in major liver diseases. Building on this foundation, we critically evaluate the evolving landscape of macrophage-targeted therapies. Finally, we discuss persistent translational challenges and future directions essential for achieving precise, context-dependent manipulation of macrophage biology in clinical practice.

## 2 Biological basis of liver macrophages: origin, heterogeneity, and plasticity

The diversity of liver macrophages stems from their developmental origins and microenvironmental shaping. Tissue-resident KCs originate from embryonic yolk sac and fetal liver hematopoietic precursors and are maintained postnatally by local proliferation. They express unique markers such as C-type lectin domain family 4 member F (CLEC4F) and T cell immunoglobulin and mucin domain containing protein 4 (TIM4), adhere to hepatic sinusoid endothelial cells, and are particularly enriched in periportal regions. KCs are instrumental in maintaining immune tolerance and serve as the first line of defense against pathogens and antigens from the portal blood (19, 20).

MoMFs are derived from circulating monocytes recruited to the injured liver via chemokine ligand-receptor axes. Their differentiation fate is precisely regulated by complex signaling networks within the injury microenvironment. For instance, signals like IFN-γ and GM-CSF drive polarization towards a classically activated (M1) phenotype, producing pro-inflammatory cytokines (e.g., IL-1β, TNF-α) that exacerbate tissue damage. In contrast, IL-4 and IL-13 promote an alternatively activated (M2) phenotype, characterized by the secretion of IL-10 and TGF-β, which facilitate inflammation resolution and tissue repair (21, 22).

Transcending this binary framework, single-cell technologies have unveiled a spectrum of specialized macrophage subsets with distinct identities and functions. These newly defined subsets—such as lipid-associated macrophages (LAMs)—are maintained and undergo transitions between states under the governance of multiple mechanisms, including transcription factors and metabolic reprogramming (23, 24). Beyond cell-intrinsic programming, soluble factors in the microenvironment provide crucial extrinsic instructions. A prime example is glutamate, released from stressed hepatocytes, which can metabolically reprogram macrophages into a potent pro-regenerative state, defining a novel form of intercellular metabolic crosstalk (25). Importantly, the nature of the cellular microenvironment itself also provides critical cues. Recent evidence introduces the identity of the apoptotic cells being engulfed as a key determinant. For instance, in an IL-4-rich setting, macrophages that phagocytose apoptotic neutrophils acquire a tissue-remodeling gene signature, whereas those that ingest apoptotic hepatocytes adopt a tolerogenic phenotype. This highlights that the specific “meal” of efferocytic macrophages can actively instruct their functional polarization, adding a novel layer of complexity to their plasticity (26).

Beyond upon these cell-intrinsic and microenvironmental programming mechanisms, the two macrophage populations engage in constant bidirectional crosstalk that fundamentally shapes their identity and function throughout disease. Resident KCs initiate and regulate the hepatic immune niche by producing C-C motif chemokine ligand 2 (CCL2) to recruit C-C motif chemokine receptor 2 (CCR2)^+^ monocytes and secreting IL-1β/TNF-α that shape recruited macrophages polarization following injury (27–29). Consequently, KCs depletion impairs these MoMFs response (21, 30). Conversely, MoMFs infiltration profoundly reshapes the KCs compartment (31, 32). In addition to competing for survival signals like CSF-1 (9, 33, 34), infiltrating monocytes actively replace embryonic KCs (EmKCs) during injury (35–37). For example, in cholestatic and toxic models, recruited Ly6C^+^ monocytes differentiate into monocyte-derived KCs (MoKCs) that, due to enhanced proliferation and anti-apoptotic properties, rapidly dominate the KC pool. Crucially, this replacement represents functional upgrading, not mere substitution. MoKCs exhibit a distinct transcriptional repertoire characterized by enhanced expression of genes for tissue repair, matrix remodeling, and phagocytosis, translating into stronger phagocytic capacity and greater ability to attenuate liver fibrosis *in vivo (*38). Understanding all these fundamental biological properties is essential for deciphering their functional diversity in disease.

## 3 Dynamic macrophage transitions in acute liver injury and repair: temporal functional evolution from pro-inflammatory to reparative

Acute liver injury, while clinically severe, offers a powerful temporal paradigm for deciphering the functional plasticity of macrophages. In such settings, the sequence of tissue damage, inflammation, and regeneration unfolds over a compressed timeline, allowing for precise dissection of macrophage roles at each phase. Experimental models such as acetaminophen-induced acute liver injury (APAP-ALI) and Concanavalin A (ConA)-induced autoimmune hepatitis (AIH) serve as exemplary windows into this dynamic process. These models reveal that macrophage functions are not fixed but undergo a stereotyped progression: early-responding subsets predominantly exacerbate tissue injury, while later-arriving or phenotypically switched subsets become indispensable for inflammation resolution and parenchymal repair.

### 3.1 Pro-inflammatory macrophage subsets in the early injury phase

During the initial stage, necrotic hepatocytes release large amounts of damage-associated molecular patterns (DAMPs), activating KCs and recruiting inflammatory monocytes from the circulation. The early injury phase is orchestrated by distinct pro-inflammatory subsets, the identity of which can vary depending on the injury model. In sterile injury driven by hepatocyte necrosis, such as APAP-ALI, CCR2^+^ MoMFs are rapidly recruited, forming dense clusters around necrotic areas. Notably, these macrophages also promote neutrophil infiltration, establishing a collaborative pro-inflammatory network that amplifies tissue damage. The work of Mossanen et al. clearly demonstrated the crucial destructive role of this population within the first 24 hours post-injury (39). Conversely, in immune-mediated injury like ConA-induced AIH, a bone marrow-derived CD169^+^ macrophage subset plays a pivotal role. Activated by IFN-γ, these cells secrete CCL12 to recruit additional CCR2^+^ monocytes, establishing a feed-forward loop of inflammation (40). Furthermore, the pro-inflammatory switch in macrophages is regulated by critical post-translational modifications. In AIH, TNF-α induces caspase-8-dependent cleavage of the deubiquitinase cylindromatosis (CYLD) within macrophages, thereby relieving its suppression of the MEK1/2-ERK signaling pathway. This renders macrophages hyperresponsive to damage signals (e.g., S100A9), leading to excessive production of neutrophil chemokines and driving liver injury. This CYLD-MEK1/2 axis represents a core mechanism of macrophage dysfunction (41). Aforementioned findings underscore the theoretical rationale for targeting early inflammatory recruitment. However, whether all these pro-inflammatory subsets are entirely detrimental remains unclear, as some evidence suggests that a controlled inflammatory response may be necessary to initiate repair (42, 43).

### 3.2 Reparative macrophage subsets in the resolution phase

As the injury enters the resolution phase, macrophage-led repair programs are initiated. Several subsets with reparative functions have been identified.

#### 3.2.1 MerTK^+^ macrophages

During the recovery phase of APAP-ALI, the number of Mer tyrosine kinase (MerTK)^+^ macrophages increases significantly. They efficiently clear apoptotic/necrotic cells by recognizing “eat-me” signals like phosphatidylserine. This process not only removes the source of damage but also actively suppresses inflammation via downstream signaling (44). Intriguingly, the functional reprogramming following efferocytosis may extend to the metabolic recycling of the engulfed cargo. Pioneering work reveals that macrophages phagocytosing dead—but not live—bacteria utilize derived nutrients to fuel the synthesis of protective metabolites like glutathione and itaconate. This process is governed by a bacterial viability-sensing pathway: dead cargo-derived cyclic adenosine monophosphate (cAMP) sustains the intracellular adenosine monophosphate (AMP) pool, activating AMP-activated protein kinase (AMPK) and inhibiting mechanistic target of rapamycin complex C1 (mTORC1), thereby orchestrating a metabolic shift towards antioxidant and anti-inflammatory responses. This paradigm posits that signals from dead cells can be metabolically sensed to fundamentally skew macrophage function toward a repair phenotype (45).

Complementing this rapid functional rewiring, efferocytosis itself can trigger macrophage proliferation through a defined DNase2a–DNA-PKcs–mTORC2–Myc pathway — a process termed efferocytosis-induced macrophage proliferation (EIMP). This expands the pool of reparative macrophages, enhancing their capacity for continual apoptotic cell clearance and tissue resolution, and may underlie the sustained reparative capacity exhibited by MerTK^+^ macrophages (46). The existence and protective role of this subset have been validated in APAP-induced acute liver failure (AALF) patients (44).

#### 3.2.2 CXCR4^+^ macrophages

Nguyen et al. demonstrated that hepatocytes promote C-X-C motif chemokine receptor 4 (CXCR4) expression on macrophages during repair. Further research indicated that these CXCR4^+^ macrophages are key regulators of inflammation resolution. CXCR4 expression triggers their own apoptotic program, facilitating the timely exit of infiltrating immune cells, which is crucial for complete inflammation resolution and restoration of hepatic homeostasis (47).

#### 3.2.3 TREM2^+^ LAMs

Triggering receptor expressed on myeloid cells 2 (TREM2), an immunoglobulin superfamily receptor, plays a key role in lipid metabolism and phagocytosis. De Ponti et al., using single-nucleus RNA sequencing, identified TREM2^+^ LAMs and LAM-like KCs in the recovery phase of APAP injury. Enriched around central veins, they highly express lipid metabolism-related genes (e.g., *Lpl*, *Cd36*) and exhibit superior phagocytic clearance capacity (48).

#### 3.2.4 MARCO^+^ macrophages

Poole et al. found that macrophage receptor with collagenous structure (MARCO)^+^ macrophages may attenuate liver injury by boosting the expression of pro-resolution genes (e.g., *MerTK*, *Gpnmb*) and regulating the number of CD11c^+^ repair cells (49).

#### 3.2.5 Reparative subsets in the ConA model

Feng et al. revealed the synergistic action of two MoMFs subsets during ConA injury resolution. *C1q*^+^ MoMFs clear necrotic tissue via complement-mediated phagocytosis, while *Pdgfb*^+^ MoMFs secrete platelet-derived growth factor (PDGF) to activate hepatic stellate cells (HSCs), generating contraction signal that help squeeze and eliminate necrotic areas (21). Additionally, Zeng et al. reported that the *IRG1*/itaconate/Nrf2 axis upregulates TIM4 expression on macrophages, enhancing their clearance of apoptotic cells and promoting tissue repair (50).

Earlier studies identified a conserved metabolic circuit essential for the high efferocytic activity of reparative macrophages. This pathway involves the conversion of apoptotic cell-derived arginine and ornithine into putrescine via arginase 1 (Arg1) and ornithine decarboxylase (ODC). Putrescine, in turn, fuels a self-reinforcing cycle that promotes continual efferocytosis—the sequential clearance of apoptotic corpses—by enhancing Rac1-dependent phagosome internalization (51). Thus, metabolic processing of one apoptotic cell augments the capacity to clear the next, providing a fundamental mechanism for injury resolution. However, the key extracellular signals that initiate this functional state in macrophages remain a critical unknown.

## 4 Multifaceted roles of macrophages in chronic liver diseases

In stark contrast to the defined temporal sequence observed in acute injury, chronic liver diseases confront macrophages with a sustained and multifaceted assault. Persistent exposure to a milieu of inflammatory, metabolic, and fibrogenic signals imposes a state of prolonged polarization pressure, leading to a co-existence of functionally divergent, and often opposing, macrophage subsets. This heterogeneity is not merely a passive reflection of disease pathology but actively steers the long-term trajectory—determining whether the liver progresses toward fibrosis and failure or maintains a precarious equilibrium. This section examines the dualistic nature of macrophage responses in three major chronic disease contexts: chronic hepatitis B virus (HBV), metabolic dysfunction-associated steatotic liver disease and/or steatohepatitis (MASLD/MASH), and progressive fibrosis/cirrhosis.

### 4.1 Hepatitis B virus infection: fine-tuning of immune responses

In HBV infection, macrophages face the dual challenge of exerting antiviral activity while curbing immunopathology. This balance is reflected in the expansion of distinct, functionally opposed subsets.

#### 4.1.1 Pro-inflammatory/pathogenic subsets

Research has focused on CD205^+^ and CD206^+^ macrophages. In hepatitis B surface antigen (HBsAg)-transgenic mice, CD205^+^ macrophages are hypersensitive to bacterial products from the gut (e.g., CpG oligodeoxynucleotide (CpG ODN)). Upon stimulation, they produce excessive IL-12, activating NKT cells to highly express FasL. This binds to Fas upregulated on hepatocytes, triggering massive hepatocyte apoptosis and severe liver injury (52). Importantly, this pro-inflammatory CD205^+^ macrophage subset is also expanded in the livers of patients with chronic hepatitis B (CHB). These human CD205^+^ macrophages exhibit an activated phenotype (elevated HLA-DR, CD14, and CD16) and produce higher levels of inflammatory cytokines such as TNF-α and IL-12, underscoring their likely contribution to sustained inflammation and liver injury in the clinical setting of HBV infection (53). Another study in patients with virus-related (HBV/Hepatitis C virus (HCV)) cirrhosis found an expanded intrahepatic CD14^+^HLA-DR^hi^CD206^+^ macrophage subset with defective endotoxin tolerance. Upon persistent exposure to gut microbial products, instead of downregulating inflammation, they continuously secrete TNF-α and GM-CSF, driving chronic liver inflammation (54).

#### 4.1.2 Protective/antiviral subsets

Wang et al. identified a population of liver-sessile CD3^+^ macrophages emerging during the clearance phase of acute HBV infection. Their generation depends on virus-specific T cells and anti-HBsAg antibodies. Besides possessing strong phagocytic ability, they exhibit direct cytotoxic functions, thereby accelerating viral clearance (55).

### 4.2 Metabolic dysfunction-associated steatotic liver disease: central regulators of inflammation and fibrosis

MASLD and its progressive form, MASH, represent typical scenarios of macrophage dysfunction, where a lipotoxic microenvironment shapes unique macrophage subsets.

#### 4.2.1 Disease drivers:

##### 4.2.1.1 S100A4^+^ MoMFs

In MASLD, downregulation of the m6A methyltransferase METTL14 leads to reduced expression of glutaminase 2 (GLS2). This metabolic alteration promotes oxidative stress, which in turn activates the myeloid differentiation primary response 88 (MyD88)/NF-κB pathway in monocytes. This signaling cascade drives the expansion of a pathogenic S100A4^+^ macrophage subset that secretes pro-inflammatory factors and activates HSCs, thereby linking steatosis to inflammation and fibrosis (56).

##### 4.2.1.2 Hepatic lipid-associated macrophages

Induced by long-chain fatty acids and dependent on Egr2, CD11c^hi^Mincle^hi^ hLAMs likely aggravate MASH by producing cytokines (e.g., TNF-α) and activating HSCs (57).

##### 4.2.1.3 Periportal CD74^+^ LAMs

Adding a spatial dimension to this heterogeneity, studies reveal a periportal THRSP/PA/MIF/CD74 axis. Here, hepatocyte-derived thyroid hormone-responsive protein (THRSP) promotes palmitic acid (PA) and macrophage migration inhibitory factor (MIF) secretion to recruit CD74^+^ LAMs, creating a localized pro-inflammatory and pro-fibrogenic niche (58).

#### 4.2.2 Metabolic protectors

##### 4.2.2.1 OPN^+^ macrophages/*Spp1*^hi^ macrophages

Macrophage-derived osteopontin (OPN, *SPP1*) exerts protective effects in MASLD. Han et al. elucidated that OPN upregulates oncostatin-M (OSM) signaling in hepatocytes, enhancing arginase 2 (Arg2) expression and promoting fatty acid oxidation, thereby reducing hepatic lipid accumulation (59).

##### 4.2.2.2 CD209a-expressing macrophages

By modulating the signal transducer and activator of transcription 3 (STAT3) signaling pathway, this subset can suppress liver inflammation and ameliorate steatosis (60). In addition to transcriptional regulation, receptor crosstalk on the macrophage surface can also fine-tune their function. For instance, DC-SIGN (CD209) can bind Toll-like receptor 4 (TLR4) and promote its internalization, selectively attenuating MyD88-dependent pro-inflammatory signaling (e.g., NF-κB) while enhancing the TBK1-IRF3 pathway. This reprogramming of TLR4 signaling shifts DC-SIGN^+^ macrophages toward an anti-inflammatory secretory profile (e.g., increased IL-10), thereby contributing to their protective role in MASLD models (61).

##### 4.2.2.3 Notch signaling-mediated switching

Guo et al. provided a dynamic perspective: blocking the Notch-Recombination signal binding protein for immunoglobulin kappa J region (RBPJ) signal in macrophages not only inhibits the differentiation of Ly6C^hi^ monocytes into inflammatory macrophages but also promotes their conversion to reparative Ly6C^lo^ monocytes. The latter enhance lipid uptake via upregulated CD36 and exert endothelial protective effects, thereby ameliorating MASH (62).

##### 4.2.2.4 PHGDH-mediated intrinsic metabolic checkpoint

Moreover, intrinsic metabolic reprogramming within macrophages directly regulates their function. It has been established that the serine synthesis enzyme phosphoglycerate dehydrogenase (PHGDH) acts as a critical intracellular metabolic checkpoint (63). Specifically, under lipotoxic stress, tetrameric PHGDH directly binds and inhibits transforming growth factor-β-activated kinase 1 (TAK1), thereby blocking the NF-κB/MAPK inflammatory cascade. Myeloid specific PHGDH deficiency exacerbates diet induced steatohepatitis, whereas its overexpression confers protection, nominating PHGDH as a novel target for metabolic immune precision therapy in MASLD (64).

### 4.3 Liver fibrosis and cirrhosis: major regulators of extracellular matrix metabolism

In fibrosis, macrophages are primary regulators of excessive extracellular matrix (ECM) deposition but also harbor pro-resolution potential.

#### 4.3.1 Pro-fibrogenic subsets

##### 4.3.1.1 *CD9*^+^*TREM2*^+^ macrophages

Fabre et al. identified a pro-fibrogenic subset induced by type III inflammatory cytokines (GM-CSF, IL-17A) and prevalent in human and mouse fibrotic tissues. They activate mesenchymal cells and promote type I collagen deposition, acting as a central driver of fibrosis (65).

##### 4.3.1.2 Nogo-B^+^ macrophages

Zhang et al. found that in liver fibrosis, macrophages highly expressing neurite outgrowth inhibitor protein B (Nogo-B) recruit the deubiquitinase USP14 to stabilize receptor-interacting serine-threonine kinase 3 (RIRP3), thereby activating the NOD-like receptor protein 3 (NLRP3) inflammasome and inducing necroptosis, which drives fibrosis (66).

##### 4.3.1.3 Tet2-deficient pro-inflammatory macrophages

Recent research revealed how age-related somatic mutations can drive fibrosis. Tet2 gene-deficient clonal hematopoiesis, common in the elderly, drives the abnormal expansion of MoMFs via positive feedback loops like CCL2/CCR2. These macrophages secrete IL-6 to activate HSCs, accelerating fibrosis (67).

#### 4.3.2 Pro-repair/regenerative subsets

In patients and mouse models with decompensated cirrhosis undergoing splenectomy, Yang et al. discovered an increased population of CD300E^+^ MoMFs. They secrete nicotinamide phosphoribosyltransferase (NAMPT), elevating NAD^+^ levels in hepatocytes and promoting their proliferation and regeneration (68).

Collectively, macrophages exhibit profound heterogeneity and functional duality across acute and chronic liver diseases. To provide a systematic overview of the key macrophage subsets discussed, their phenotypes and principal functions in diverse disease contexts are summarized in **Table 1**.

**Table 1:** Macrophage subsets’ role in diverse liver diseases.

Disease	Macrophage phenotypes	Mouse model/Patient	Main functions		Reference
APAP-ALI	CCR2^+^ Mφs	APAP-ALI mouse model Patient with AALF	deleterious	Infiltrate injured liver and form necrotic cluster Exacerbate early injury via proinflammatory functions	Mossanen et al.(2016) (39)
	MerTK^+^ Mφs	APAP-ALI mouse model Patient with AALF	reparative	Suppress innate and enhance efferocytic/ phagocytic responses	Triantafyllou et al.(2018) (44)
	CXCR4^+^ Mφs	APAP-ALI mouse model		Trigger their cell death via apoptosis at the end of the recovery phase	Nguyen et al.(2022) (47)
	TREM2^+^ LAMs LAM-like KCs	APAP-ALI mouse model Patient with APAP-ALI		Clear dying cells and prevent exacerbated fibrosis	De Ponti et al.(2025) (48)
	MARCO^+^ Mφs	APAP-ALI mouse model		Boost pro-resolution gene expression and necrotic clearance Regulate CD11c^+^ repair cells	Poole et al.(2025) (49)
AIH	CD169^+^ Mφs	ConA induced acute liver injury mouse model Patient with AIH	deleterious	Secrete CCL12 in response to IFN-γ to recruit CCR2^+^ Monocytes	Li et al.(2025) (40)
	*C1q*^+^ Mφs *Pdgfb*^+^ Mφs	ConA induced acute liver injury mouse model	reparative	Phagocytose necrotic tissues Activate HSCs to contract and squeeze necrotic areas	Feng et al.(2023) (21)
HBV infection	CD205^+^ Mφs	CpG-ODN induced acute liver injury mouse model Patient with CHB	deleterious	Produce excessive IL-12 and drive NKT cells-mediated hepatocytotoxicity through Fas signaling pathway	Hou et al.(2016) (52), Yong et al.(2017) (53)
	CD206^+^ Mφs	HBV-infected humanised mouse model Patient with viral-related liver cirrhosis and/or HCC		Driven by gut-derived bacterial products, generate TNF-α and GM-CSF, contributing significantly to sustained liver inflammation	Tan-Garcia et al. (2017) (54)
	CD3^+^ Mφs	HBV fast-cleared mouse model Patient with AHB	reparative	Perform phagocytosis and cytotoxic functions	Wang et al.(2024) (55)
MASLD/MASH	S100A4^+^ Mφs	HFD induced MASLD mouse model	deleterious	Secrete inflammatory cytokines and activate HSCs	Wang et al.(2024) (56)
	hLAMs(CD11c^hi^Mincle^hi^)	CDA-HFD induced MASH mouse model		Produce more inflammatory cytokines (TNF-α, IL-6)	Iwata et al.(2024) (57)
	*Spp1*^hi^ Mφs	HFFC diet induced MASH mouse model	reparative	Upregulate oncostatin-M, thereby enhancing fatty acid oxidation	Han et al.(2023) (59)
	Ly6c^hi^ Monos → Ly6c^lo^ Monos	MCDD/WD induced MASH mouse model		Enhance lipid uptake via upregulated CD36 and strengthen endothelial protection	Guo et al.(2024) (62)
	DC-SIGN^+^ Mφs	HFHC diet induced MASH mouse model Patient with MASLD		Facilitate macrophage TLR4 endocytosis Suppress MyD88-dependent pro-inflammatory responses	Liu et al.(2025) (61)
Liver fibrosis/cirrhosis	*CD9*^+^*TREM2*^+^ Mφs	Multiple mouse fibrosis model Patient with NASH or IPF	deleterious	Activate mesenchymal cells and promote collagen | deposition	Fabre et al.(2023) (65)
	Nogo-B^+^ Mφs	Multiple mouse fibrosis model Patient with liver fibrosis		Facilitate NLRP3 inflammasome activation and induce necroptosis	Zhang et al.(2025) (66)
	CD300E^+^ Mφs	CCl_4_-induced cirrhosis mouse model with splenectomy patient with decompensated cirrhosis after splenectomy	reparative	Facilitate hepatocyte NAD^+^ synthesis via the secretion of NAMPT to promote hepatocyte proliferation	Yang et al.(2025) (68)

APAP-ALI, acetaminophen-induced acute liver injury; AIH, autoimmune hepatitis; HBV, hepatitis B virus; MASLD, metabolic dysfunction-associated fatty liver disease; Mφs, macrophages; TREM2, trigger receptor expressed on myeloid cells 2; LAMs, lipid-associated macrophages; MARCO, macrophage receptor with collagenous structure; hLAMs, hepatic lipids-associated macrophages; Monos, monocytes; Nogo-B, neurite outgrowth inhibitor protein B; AALF, APAP-induced acute liver failure; ConA, Concanavalin A; CpG-ODN, CpG oligodeoxynucleotide; CHB, chronic hepatitis B; HCC, hepatocellular carcinoma; AHB, acute hepatitis B; HFD, high-fat diet; CDA-HFD, choline-deficient amino acid-defined high-fat diet; MASH, metabolic dysfunction-associated steatohepatitis; HFFC, high fat, high fructose and high cholesterol; MCDD, methionine and choline-deficient diet; WD, western diet; HFHC, high-fat, high-cholesterol; CCl_4_, carbon tetrachloride; NASH, non-alcoholic steatohepatitis; IPF, idiopathic pulmonary fibrosis; HSCs, hepatic stellate cells; NAMPT, nicotinamide phosphoribosyltransferase.

## 5 Macrophage-targeted therapeutic strategies: from broad suppression to precise modulation

**Figure 1:**
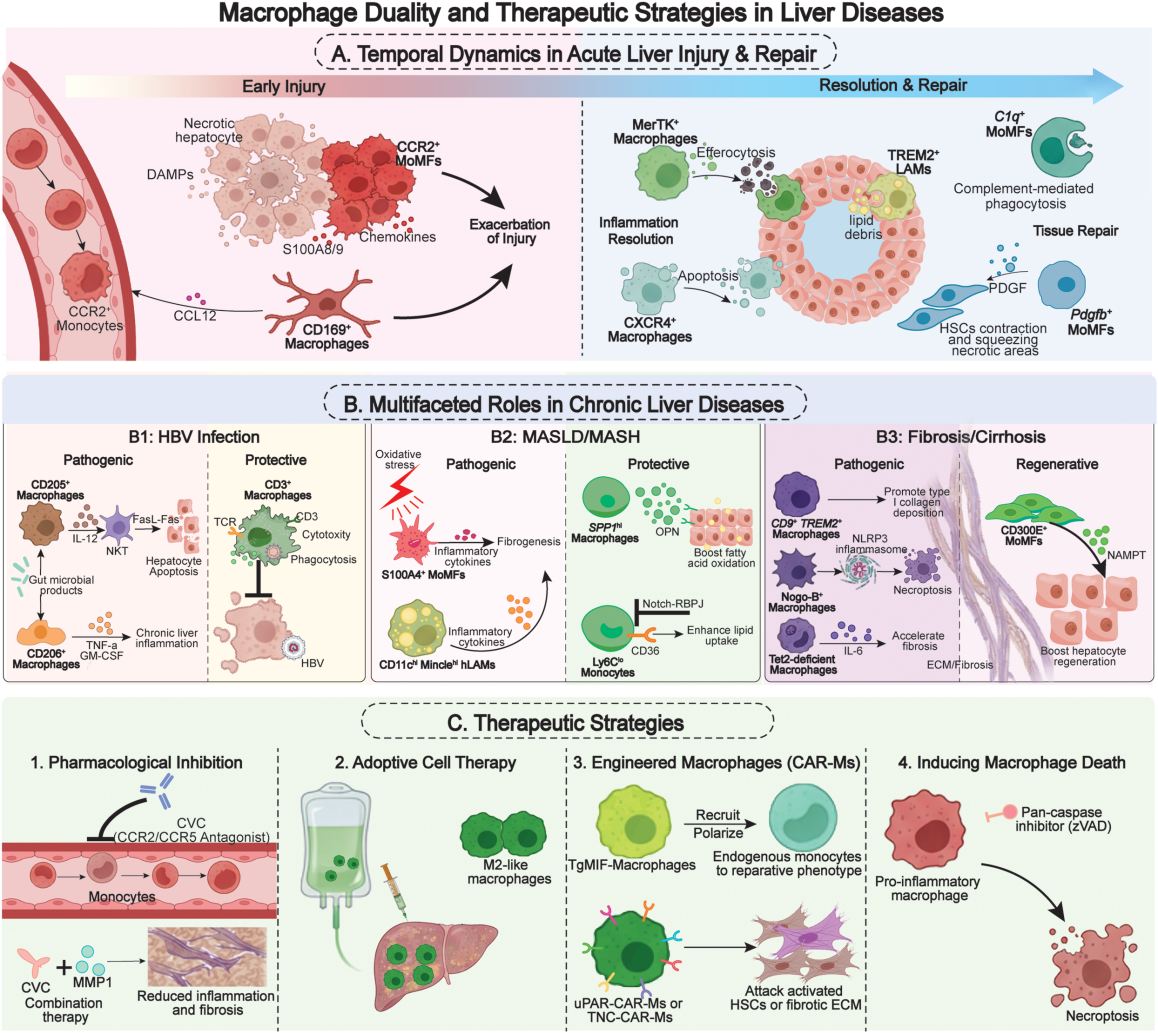
Macrophage duality and therapeutic strategies in liver diseases. **(A, B)** Schematic overview of functionally distinct macrophage subsets in acute and chronic liver disease. Pro-inflammatory/pathogenic subsets (Foes, left) and pro-resolving/reparative subsets (Friends, right) are shown with their phenotypic markers and key functions. Their temporal shift or coexistence dictates outcome. **(C)** Evolution of macrophage-targeted therapeutic strategies. From left to right: pharmacological inhibition (e.g., CCR2/5 antagonist); adoptive transfer of reparative subsets (e.g., AAMs); engineered cell therapies (e.g., CAR-Ms) and inducing macrophage death. MoMFs, monocyte-derived macrophages; DAMP, damage-associated molecular pattern; TREM2, triggering receptor expressed on myeloid cells 2; LAMs, lipid-associated macrophages; PDGF, platelet-derived growth factor; HSCs, hepatic stellate cells; IL, interleukin; TNF, tumor necrosis factor; GM-CSF, granulocyte-macrophage colony stimulating factor; TCR, T cell receptor; HBV, hepatitis B virus; OPN, osteopontin; Notch-RBPJ, Notch-Recombination signal binding protein for immunoglobulin kappa J; NLRP3, NOD-like receptor protein 3; ECM, extracellular matrix; NAMPT, nicotinamide phosphoribosyltransferase; CVC, cenicriviroc; MMP1, matrix metalloproteinase 1; CAR-Ms, chimeric antigen receptor macrophages; TgMIF, *Toxoplasma gondii* macrophage migration inhibitory factor; uPAR, urokinase plasminogen activator receptor; TNC, tenascin-C.

Building upon the detailed understanding of macrophage heterogeneity and functional duality in liver diseases, therapeutic efforts have evolved from a simplistic paradigm of broad anti-inflammatory suppression toward more nuanced, subset-specific interventions. This conceptual shift—from unselective targeting to subset-specific modulation—is schematically summarized in **Figure 1**, which integrates the dual roles of hepatic macrophage subsets across disease contexts with the evolving landscape of precision therapeutic strategies.

The overarching goal is no longer to indiscriminately eliminate or silence all macrophages, but to strategically modulate their composition and function: suppressing or removing pathogenic subsets while preserving, enhancing, or even delivering reparative ones.

### 5.1 Pharmacological inhibition: challenges and combination strategies

A classic example is the CCR2/CCR5 dual antagonist Cenicriviroc (CVC). In the AURORA phase III trial, CVC monotherapy failed to significantly alleviate fibrosis in nonalcoholic steatohepatitis (NASH) patients (69). This outcome prompts reflection: merely blocking monocyte recruitment may be insufficient to reverse established fibrotic networks. It suggests the future need for combination therapies. Preclinical work by Geervliet et al. showed that combining CVC with matrix metalloproteinase 1 (MMP1, an enzyme that degrades ECM) synergistically reduced inflammation and fibrosis, combining inhibition of new recruitment with promotion of matrix degradation (28). Targeting specific pathogenic pathways in combination shows greater promise. For instance, in a Tet2-deficiency-driven liver fibrosis model, combined application of a CCL2/CCL8 inhibitor and an IL-6 neutralizing antibody synergistically inhibited monocyte recruitment and blocked their HSCs-activating signal, more effectively attenuating fibrosis than either agent alone (67).

### 5.2 Cell therapy: adoptive transfer of “M2-like” subsets and engineered macrophages

#### 5.2.1 Adoptive transfer of “M2-like” subsets

##### 5.2.1.1 Preclinical evidence

In APAP-ALI mouse models, infusion of mouse or human alternatively activated macrophages (AAMs) has been proven effective in reducing necrosis and stimulating hepatocyte regeneration. Importantly, it remains effective beyond the therapeutic window of N-acetylcysteine (NAC) (70, 71). Similarly, in liver fibrosis mouse models, infusion of bone marrow-derived macrophages (BMDMs) or macrophages induced by IL-34-conditioned culture mitigated fibrosis by increasing collagen degradation and inducing HSC apoptosis, among other mechanisms (72, 73).

##### 5.2.1.2 Clinical translation progress

A phase I trial (Moroni et al., 2019) first demonstrated that reinfusing autologous monocyte-derived macrophages (MDMs) into cirrhosis patients is safe and led to observed improvements in Model for End-Stage Liver Disease (MELD) score and liver stiffness (74). However, a subsequent phase II randomized controlled trial (Brennan et al., 2025) failed to meet its primary endpoint (difference in MELD score change at 90 days). Nonetheless, the treatment group showed good safety and an increase in serum anti-inflammatory cytokines (IL-10, IL-13) (75). These clinical trial results highlight core bottlenecks in translating reparative subset infusion therapy: cell source (autologous vs. allogeneic), standardization of *in vitro* expansion and polarization, product stability (e.g., functional maintenance after freeze-thaw), *in vivo* survival and phenotypic stability post-infusion, and how to precisely select treatment timing and patient populations. These clinical trial results highlight core bottlenecks, particularly the challenge of maintaining a stable reparative phenotype *in vivo* after infusion. To address this issue of phenotypic drift, novel pre-conditioning strategies are being explored. For instance, the concept of “selective macrophage feeding” — pre-exposing macrophages *in vitro* to specific apoptotic cells (e.g., apoptotic neutrophils) — can reprogram them toward a durable tissue-repair profile before transfer (26). Mechanistically, this approach can harness pathways such as EIMP, which stabilizes a reparative phenotype via the DNase2a-DNA-PKcs-mTORC2-Myc axis (46). Leveraging such mechanisms ex vivo may improve the functional persistence and therapeutic potency of adoptively transferred macrophages.

#### 5.2.2 Engineered macrophage therapy: frontiers and progress

Cellular immunoengineering provides a new paradigm for creating “smart” therapeutic macrophages, with the core aim of endowing cells with precise functions beyond natural phenotypes. For example, transgenic macrophages expressing *Toxoplasma gondii* macrophage migration inhibitory factor (TgMIF) can systemically recruit and polarize endogenous monocytes/macrophages towards a reparative phenotype with low inflammation, thereby remodeling the microenvironment (76). Chimeric antigen receptor macrophages (CAR-Ms) targeting the urokinase plasminogen activator receptor (uPAR) not only clear activated HSCs but also act as antigen-presenting cells to activate endogenous T cells, synergistically combating fibrosis (77). Furthermore, CAR-Ms targeting the extracellular matrix protein Tenascin-C (TNC) alleviate fibrosis by clearing activated HSCs, promoting M2 macrophage infiltration, and activating CD8^+^ T cells (78). Shifting the paradigm from ex vivo engineering, a complementary approach exploits the inherent homing ability of GATA6^+^ Large Peritoneal Macrophages (GLPMs) to sites of acute liver injury. By loading these endogenous macrophages with therapeutic nanoparticles (e.g., containing high mobility group box 1 (HMGB1) siRNA), they can be repurposed as “living delivery systems,” achieving targeted drug enrichment and potent anti-inflammatory effects (79).

Another innovation involves signal-converting CAR-Ms that reprogram inflammatory cues into repair signals. These engineered macrophages are designed to bind TNF-α but activate the IL-4/STAT6 pathway, thereby adopting a reparative phenotype selectively within inflamed tissue (e.g., liver ischemia-reperfusion injury) and autonomously reverting to a resting state once inflammation subsides. This self-limiting, microenvironment-sensing strategy represents a significant step toward intelligent, context-responsive therapies for liver disease (80).

### 5.3 Inducing macrophage death: an emerging intervention concept

Selectively eliminating pathogenic macrophages may constitute a third frontier strategy. In an AIH model, the pan-caspase inhibitor Z-Val-Ala-DL-Asp-fluoromethylketone (zVAD) upregulated autocrine IL-10 in macrophages, increased tumor necrosis factor receptor 1 (TNFR1) expression, sensitizing them to necroptosis, ultimately leading to the death of these pro-inflammatory macrophages (81). This reveals a new logic: using drugs to reprogram macrophage death sensitivity, thereby “clearing” pathogenic cells at the inflammatory site.

The evolving landscape of macrophage-targeted therapies, spanning pharmacological inhibition to innovative cell-based approaches, underscores a shift towards precision modulation. **Table 2** compiles key preclinical and clinical studies of macrophage-based cell therapies, detailing the cell types used, disease models, core therapeutic effects, and clinical translation stages.

**Table 2:** Preclinical/clinical trials of macrophage-based cell therapy.

Disease	Cell Types	Mouse model/Patient	Core Efficacy	Key Limitations/Challenges	Clinical Stage	Reference
APAP-ALI	Mouse AAMs human AAMs	APAP-ALI mouse model	Reduce hepatocellular necrosis, HMGB1 translocation and neutrophils infiltration Stimulate hepatocytes proliferation	Species barriers and rapid clearance Logistical challenge for emergency use	Preclinical	Starkey Lewis et al. (2020) (70)
	Cryopreserved hAAMs	APAP-ALI mouse model	Mitigate liver necrosis and modulate Inflammatory responses Effective beyond NAC window	Impact of cryopreservation Lack of traditional biomarkers response	Preclinical	Candela et al. (2025) (71)
Liver Fibrosis/ Cirrhosis	Mouse BMDMs	Multiple mouse fibrosis model	Increase collagen degradation and HSCs apoptosis	Potential safety concerns Reliance on complex host immune modulation	Preclinical	Ma et al. (2017) (72)
	Mouse BMDMs Human MDMs	Multiple mouse fibrosis model NSG mouse with GVHD	Suppress liver inflammation, HSCs and T cell activation	Unclear superiority over CSF-1 Mφs Humanized mouse model limitations	Preclinical	Igarashi et al. (2025) (73)
	Autologous MDMs	9 cirrhosis patients (MELD 10-16)	Safe (no DLT) Reduce MELD scores and liver stiffness	*In vivo* phenotypic stability Patient-to-patient variability Scalability	Phase I ISRCTN10368050	Moroni et al. (2019) (74)
	Autologous MDMs	51 cirrhosis patients (MELD 10-17)	Failure to meet the primary endpoint(improvement in MELD score at 90 days) No liver-related severe adverse events or deaths		Phase II RCT ISRCTN10368050	Brennan et al. (2025) (75)
	TgMIF-transgenic Mφs (Engineered Mφs)	Multiple mouse fibrosis model	Recruit/polarize endogenous mφs toward a restorative phenotype	Potential for on-target/off-tumor toxicity Complex and costly GMP manufacturing Long-term safety unknowns	Preclinical	Chen et al.(2024) (76)
	uPAR-targeted CAR-Ms (Engineered Mφs)	Multiple mouse fibrosis model	Exert direct phagocytic activity against HSCs and elicit adaptive antifibrotic T cell responses		Preclinical	Dai et al (2024) (77)
	TNC-targeted CAR-Ms (Engineered Mφs)	Multiple mouse fibrosis model	Target fibrotic ECM via TNC clearance, inhibit pro-fibrotic signaling, and engage CD8^+^ T cell-dependent immunity		Preclinical	Chen et al(2025) (78)

APAP-ALI, acetaminophen induced acute liver injury; AAMs, alternatively activated macrophages; hAAMs, human alternatively activated macrophages; BMDM, bone marrow-derived macrophages; MDMs, monocyte-derived macrophages; TgMIF, *Toxoplasma gondii* macrophage migration inhibitory factor; Mφs, macrophages; uPAR, urokinase plasminogen activated receptor; CAR-Ms, chimeric antigen receptor macrophages; TNC, Tenascin-C; GVHD, graft versus host disease; MELD, Model for End-Stage Liver Disease; HMGB1, high mobility group box 1;NAC, N-acetylcysteine;HSCs, hepatic stellate cells; DLT, dose-limiting toxicities; ECM, extracellular matrix; CSF-1, colony stimulating factor 1; GMP, Good Manufacturing Practice.

## 6 Future perspectives: towards spatiotemporally precise macrophage medicine

Despite the significant advances detailed above, the translation of our understanding of hepatic macrophage biology into durable and precise clinical therapies remains a formidable challenge. This gap stems not from a single limitation, but from interconnected hurdles across technological resolution, biological model fidelity, and clinical translation paradigms. By consolidating the unresolved questions raised throughout this review, we can chart a path toward spatiotemporally precise macrophage medicine.

### 6.1 Unresolved questions in macrophage dynamics and interrelationships

#### 6.1.1 Mapping cellular trajectories and fates

A recurring theme across all disease contexts is our incomplete understanding of how different macrophage subsets relate to one another. Are reparative subsets like MerTK^+^ and TREM2^+^ LAMs functionally redundant, sequentially activated, or do they represent different branches of a common reparative pathway? Is the MARCO^+^ subset a precursor to MerTK^+^ macrophages? In the ConA model, do *C1q*^+^ and *Pdgfb*^+^ subsets arise simultaneously or in a specific temporal order to coordinate necrotic tissue clearance and contraction? Addressing these questions requires moving beyond static snapshots. The integration of advanced real-time intravital imaging with lineage tracing tools will be essential to dynamically visualize the migration, proliferation, death, and phenotypic switching of individual macrophages throughout the entire disease course, thereby mapping true cellular fate.

#### 6.1.2 Deciphering spatiotemporal relationships

In chronic diseases, the picture is even more complex. In HBV infection, the spatiotemporal distribution and dynamic relationship between pro-inflammatory CD205^+^ and antiviral CD3^+^ subsets during the natural history of chronic infection are largely unknown. In MASLD, most prior studies are based on endpoint analyses, lacking longitudinal single-cell dynamic maps that track the transition from simple steatosis through inflammation to fibrosis. This limits our ability to identify the key transition nodes that drive disease progression. Furthermore, research has largely focused on the local liver environment, potentially overlooking the “pre-activation” or “pre-education” effects of systemic metabolic disorders like obesity and insulin resistance on peripheral blood monocytes before they even enter the liver. In fibrosis, our knowledge is heavily skewed towards how fibrosis forms, while the role of macrophages during its regression is scarce. After removal of the causative agent, do pro-fibrogenic subsets like *CD9*^+^*TREM2*^+^ macrophages undergo functional conversion, or are they simply eliminated?

#### 6.1.3 Identifying master regulators of phenotypic switching

The key microenvironmental signals that drive the critical switch from a pro-inflammatory to a reparative phenotype remain to be fully elucidated. While emerging insights from immunometabolism, such as the sensing of dead cell-derived metabolites (e.g., cAMP/AMP) via the AMPK/mTORC1 axis, offer a groundbreaking paradigm, a critical future direction is to determine whether and how such metabolite-sensing mechanisms operate in the context of sterile liver injury to guide the differentiation and function of the reparative subsets described above.

### 6.2 Technology-driven deep profiling and model systems

To overcome these knowledge gaps, there is an urgent need for:

#### 6.2.1 High-resolution spatial mapping

Developing and integrating high-resolution spatial multi-omics technologies (e.g., combined spatial transcriptomics and proteomics) to reveal *in situ* the spatial niches of macrophage subsets across different liver lobule zones and their interaction networks with neighboring cells. This is particularly crucial for understanding localized niches like the periportal CD74^+^ LAMs in MASH.

#### 6.2.2 Bridging model systems

Overcoming the limitations of current animal models is imperative. This includes developing animal models that more closely mimic human liver disease pathophysiology (e.g., incorporating humanized immune systems or specific human genetic risk factors like TET2 mutations) and constructing liver organoid-immune cell co-culture systems as powerful platforms for dissecting intercellular crosstalk and performing high-throughput drug screening.

### 6.3 Rational pathways for clinical translation

Building on a deeper mechanistic understanding, several steps are critical for clinical progress:

#### 6.3.1 Biomarker identification

Identifying macrophage subset-specific biomarkers is crucial for patient stratification (selecting those most likely to benefit from a particular therapy) and for monitoring treatment response. For example, can we use circulating or imaging biomarkers to identify patients with a high burden of pathogenic *CD9*^+^*TREM2*^+^ macrophages who might benefit from targeted depletion?

#### 6.3.2 Durable cell products

For cell therapies, a major hurdle is ensuring the functional persistence of adoptively transferred cells in a hostile microenvironment. Can reparative subsets like CD300E^+^ macrophages survive long-term and maintain their function in the harsh milieu of advanced cirrhosis? Strategies like “selective macrophage feeding” or genetic engineering to enhance stress resistance need to be rigorously tested.

#### 6.3.3 Combination strategies

Given the complexity of chronic diseases, monotherapies are unlikely to be sufficient. Future success will likely depend on rational combination strategies, such as pairing macrophage therapy (to promote resolution) with anti-fibrotic drugs (to degrade existing scar) or with agents that modulate the systemic metabolic environment.

#### 6.3.4 Timing is everything

Future precision modulation may extend beyond targeting functional phenotypes to precisely regulating macrophage lifespan. The study on CXCR4^+^ macrophages suggests that promoting the apoptosis of pro-inflammatory macrophages that have completed their tasks could be a novel approach to promote inflammation resolution and prevent chronicity.

## 7 Conclusion

The journey to decipher the roles of liver macrophages has evolved from recognizing their simple duality to mapping a complex atlas of functionally specialized subsets, each with the capacity to profoundly influence disease outcomes. This review outlines the landscape of functionally distinct macrophage subsets across various liver diseases and examines the therapeutic rationale and preliminary clinical attempts targeting pro-inflammatory subsets or infusing reparative ones. Recent advances in engineered macrophage research further demonstrate the potential of genetically modifying macrophages to confer targeting capability, immunomodulation, and microenvironment-remodeling functions, opening new avenues for treating liver fibrosis. Yet, their clinical translation remains hampered by an incomplete understanding of subset dynamics *in vivo*, discrepancies between model systems and human disease, and the challenge of achieving durable, context-specific effects in a chronically evolving microenvironment. Therefore, the path forward demands a fundamental paradigm shift. The goal is no longer to simply “inhibit” macrophages, but to intelligently “reprogram” or “steer” the macrophage ecosystem. This will require converging next-generation tools, advanced models, and smart clinical design to develop therapies that match the dynamic adaptability of macrophages themselves. Ultimately, decoding when and how macrophages switch from “foe” to “friend”—and vice versa—will be essential for designing interventions that tip the balance toward resolution.

## Glossary

CCR: C-C motif chemokine receptor
MerTK: Mer tyrosine kinase
HBV: hepatitis B virus
MASLD: metabolic dysfunction-associated steatotic liver disease;MASH, metabolic dysfunction-associated steatohepatitis
CD: cluster of differentiation
TREM2: triggering receptor expressed on myeloid cells 2
CAR-Ms: chimeric antigen receptor macrophages
KCs: Kupffer cells
MoMFs: monocyte-derived macrophages
CLEC4F: C-type lectin 4F
TIM4: T cell immunoglobulin and mucin domain containing protein 4
CCL: C-C motif chemokine ligand
GM-CSF: granulocyte-macrophage colony stimulating factor
IL: interleukin
TNF: tumor necrosis factor
TGF: transforming growth factor
LAMs: lipid-associated macrophages
CSF-1: colony stimulating factor 1
EmKCs: embryonic KCs
MoKCs: monocyte-derived KCs
APAP-ALI: acetaminophen-induced acute liver injury
Con-A: Concanavalin A
AIH: autoimmune hepatitis
DAMP: damage-associated molecular pattern
CYLD: cylindromatosis
cAMP: cyclic adenosine monophosphate
AMP: adenosine monophosphate
AMPK: AMP-activated protein kinase
mTORC1: mechanistic target of rapamycin complex C1
PKc: protein kinase c
EIMP: efferocytosis-induced macrophage proliferation
AALF: APAP-induced acute liver failure
CXCR4: C-X-C chemokine receptor 4
MARCO: macrophage receptor with collagenous structure
PDGF: platelet-derived growth factor
HSCs: hepatic stellate cells
*IRG*1: immune response gene 1
Nrf2: nuclear factor erythroid 2-associated factor 2
Arg1: arginase 1
ODC: ornithine decarboxylase
HBsAg: hepatitis B surface antigen
CpG: cytosine-phosphate-guanine
ODN: oligodeoxynucleotide
CHB: chronic hepatitis B
HLA: human leukocyte antigen
HCV: hepatitis C virus
GLS2: glutaminase 2
MyD88: myeloid differentiation primary response 88
NF-κB: nuclear factor kappa B
hLAMs: hepatic lipid-associated macrophages
THRSP: thyroid hormone-responsive protein
PA: palmitic acid
MIF: migration inhibitory factor
OPN: osteopontin
OSM: oncostatin-M
TLR: toll-like receptor
Notch-RBPJ: Notch-Recombination signal binding protein for immunoglobulin kappa J
PHGDH: phosphoglycerate dehydrogenase
TAK1: transforming growth factor-β-activated kinase 1
Nogo-B: neurite outgrowth inhibitor protein B
RIRP3: receptor-interacting serine-threonine kinase 3
NLRP3: NOD-like receptor protein 3
NAMPT: nicotinamide phosphoribosyltransferase
CVC: cenicriviroc
NASH: non-alcoholic steatohepatitis
MMP1: matrix metalloproteinase 1
ECM: extracellular matrix
AAMs: alternatively activated macrophages
NAC: N-acetylcysteine
BMDMs: bone marrow-derived macrophages
MDMs: monocyte-derived macrophages
MELD: Model for End-Stage Liver Disease
TgMIF: *Toxoplasma gondii* macrophage migration inhibitory factor
uPAR: urokinase plasminogen activator receptor
TNC: Tenascin-C
GLPMs: GATA6⁺ Large Peritoneal Macrophages
HMGB1: high mobility group box 1
STAT6: signal transducer and activator of transcription factor 6
zVAD: Z-Val-Ala-DL-Asp-fluoromethylketone
TNFR1: tumor necrosis factor receptor 1
GMP: Good Manufacturing Practice.
